# An alcoholic extract of *Thuja orientalis* L. leaves inhibits autophagy by specifically targeting pro-autophagy PIK3C3/VPS34 complex

**DOI:** 10.1038/s41598-021-97216-4

**Published:** 2021-09-06

**Authors:** Juneyoung Jung, Yoomi Chun, Young Pyo Jang, Myung Sook Oh, Jeong Hee Kim, Joungmok Kim

**Affiliations:** 1 Department of Life and Nanopharmaceutical Sciences, Graduate School, Kyung Hee University, Seoul, 02447 Republic of Korea; 2 Department of Oral Biochemistry and Molecular Biology, School of Dentistry, Kyung Hee University, Seoul, 02447 Republic of Korea; 3 Department of Oriental Pharmaceutical Science, College of Pharmacy, Kyung Hee University, Seoul, 02447 Republic of Korea; 4 Department of KHU-KIST Converging Science and Technology, Graduate School, Kyung Hee University, Seoul, 02447 Republic of Korea

**Keywords:** Autophagy, Target identification

## Abstract

Autophagy is a lysosome-dependent degradation program to maintain cellular homeostasis in response to a variety of stressful conditions, such as long-lived or non-functional subcellular organelles, protein aggregates, nutrient limitation, and virus/bacteria infection. Accordingly, dysregulation of autophagy is closely associated with many human pathophysiological conditions, such as neurodegenerative diseases, aging, and cancer, and autophagy is highlighted as an important therapeutic target for these human diseases. In autophagy process, PIK3C3/VPS34 complex plays important roles in autophagosome biogenesis. Accumulating evidences that inhibition of PIK3C3/VPS34 complex successfully blocks autophagy make the complex as an attractive target for the development of autophagy-specific inhibitors. However, considering that various forms of PIK3C3/VPS34 complex exist and they are involved in many different cellular functions, the targeting of the pro-autophagy PIK3C3/VPS34 complex is required to specifically inhibit autophagy. To identify autophagy inhibitors targeting the pro-autophagy complex, we have performed the screening of a customized natural product library consisting of 35 herbal extracts which are widely used in the oriental medicine as anti-inflammation and/or anti-tumor reagents. We discovered that an alcoholic extract of *Thuja orientalis* L. leaves inhibits pro-autophagy complex formation by disrupting the interaction between autophagy-specific factor, ATG14L, and the complex core unit Vps34-Beclin 1 in vitro. Also, it inhibits the nutrient starvation induced autophagy and diminished pro-autophagy PIK3C3/VPS34 complex containing either ATG14L or UVRAG in several cell lines. Our results strongly suggest that *Thuja orientalis* L. leave extract functions as an autophagy-specific inhibitor not decreasing the complex activity nor the protein level, but preventing protein–protein interaction between autophagy-specific factor (ATG14L and UVRAG) and PIK3C3/VPS34 complex core unit, Vps34-Beclin 1, thereby specifically depleting the pro-autophagy complex to inhibit autophagy.

## Introduction

Macroautophagy (referred to as autophagy hereafter) is a lysosome-dependent catabolic pathway to remove dysfunctional, unnecessary, or harmful intracellular contents, such as long-lived/damaged subcellular organelles and protein aggregates, and infected pathogens, via a double-membrane vesicle, an autophagosome^1–3^. As an essential cellular homeostasis program, dysfunction of autophagy is closely associated with various pathophysiological conditions, including metabolic disorders, neurodegenerative diseases, aging, cardiomyopathy, and cancers^1,4,5^. In this sense, autophagy is believed to provide a new avenue to control human diseases^5–8^. Although autophagy has been studied by various pharmacological approaches, however, the development of autophagy-specific modulator has just begun. Most popular way for autophagy inhibition relies on the regulation of lysosomal activity^9^. Typically, Chloroquine (CQ) prevents the fusion of autophagosome with lysosome by raising lysosomal pH. However, as a lysosomotropic agent, CQ also blocks fusion of endosomes with lysosome in endocytosis^10^. 3-methyladenine (3-MA), and wortmannin are another examples of autophagy inhibitors^11,12^. They target the autophagy-initiating PIK3C3/VPS34 lipid kinase complex to block the production of phosphatidylinositol 3-phosphate (PI3P) on the initiating autophagosome membrane ^13,14^ to recruit the autophagy machinery required for autophagosome biogenesis^3,15^. However, as a broad-spectrum inhibitor of all PI3-kinase (PIK3) family members, they also diminish PIK3C1-AKT/PKB signaling pathway that may activate autophagy by inhibiting mTORC1, a well-known negative upstream regulator of autophagy. Moreover, considering that PIK3C1-AKT/PKB signaling is involved in various cellular processes^16,17^, ranging from metabolism to apoptosis, the use of 3-MA and wortmannin as autophagy inhibitors is limited. However, the observation that they successfully inhibit autophagy, although not specific, suggests that PIK3C3/VPS34 complex is a potent target for autophagy-specific inhibitors. Much efforts have been made to develop autophagy inhibitors targeting PIK3C3/VPS34 complex and several small molecules have identified to inhibit the complex activity, and concomitantly, autophagy^18–21^. However, it should be noted that PIK3C3/VPS34 complex exists in various forms and contribute a number of cellular functions, such as multivesicular body pathway, retrograde trafficking from endosomes to the Golgi, phagosome maturation, and autophagy^22,23^, depending on the subunits binding to the PIK3C3/VPS34 complex core unit, Vps34-Beclin 1. Therefore, considering of PIK3C3/VPS34 complex core unit, Vps34-Beclin1, as a platform for the various complexes with multiple functions, the inhibitors directly targeting the subunits of the complex core unit, Vps34 lipid kinase (SAR405^18^, PIK-III^19^, and Vps34-IN1^20^) or Beclin 1 (Spautin-1^21^), may switch off all PIK3C3/VPS34 complex functions, including autophagy. Among the complex family, PIK3C3/VPS34 complex containing either ATG14L or UVRAG functions as pro-autophagy complex^24,25^ and are activated upon autophagy-inducing condition, but not the others^26–28^. These results indicate that binding of the autophagy-specific factors, such as ATG14L or UVRAG, to the complex core unit, Vps34-Beclin 1, makes the resulting complex to pro-autophagy complex, therefore, the inhibition of protein–protein interaction (PPI) between these autophagy-specific factors and Beclin 1 on the complex core unit will specifically inhibit autophagy without affecting other PIK3C3/VPS34 complexes and their functions. In this aspect, we previously developed in vitro Vps34-Beclin 1-ATG14L formation assay to monitor ATG14L binding to the complex core unit, Vsp34-Beclin 1^29^. Using the in vitro assay format, we have screened a customized library consisting of 35 natural herbal extracts, which have been widely used as anti-inflammatory, anti-tumor, or anti-bacterial/viral agents in oriental medicine. As a result, an alcoholic extract of *Thuja orientalis* L. leaves was identified to inhibit Vps34-Beclin 1-ATG14L complex formation in vitro. In addition, it potently inhibits the nutrient starvation-induced autophagy in various cells, in which it specifically diminished the pro-autophagy PIK3C3/VPS34 complex by disrupting ATG14L (or UVRAG) binding to the complex core unit, Vps34-Beclin 1.

## Results

### A screening of natural herbal medicinal extracts disrupting pro-autophagy PIK3C3/VPS34 complex by inhibiting ATG14L binding to the complex core unit, Vps34-Beclin 1

**Figure 1:**
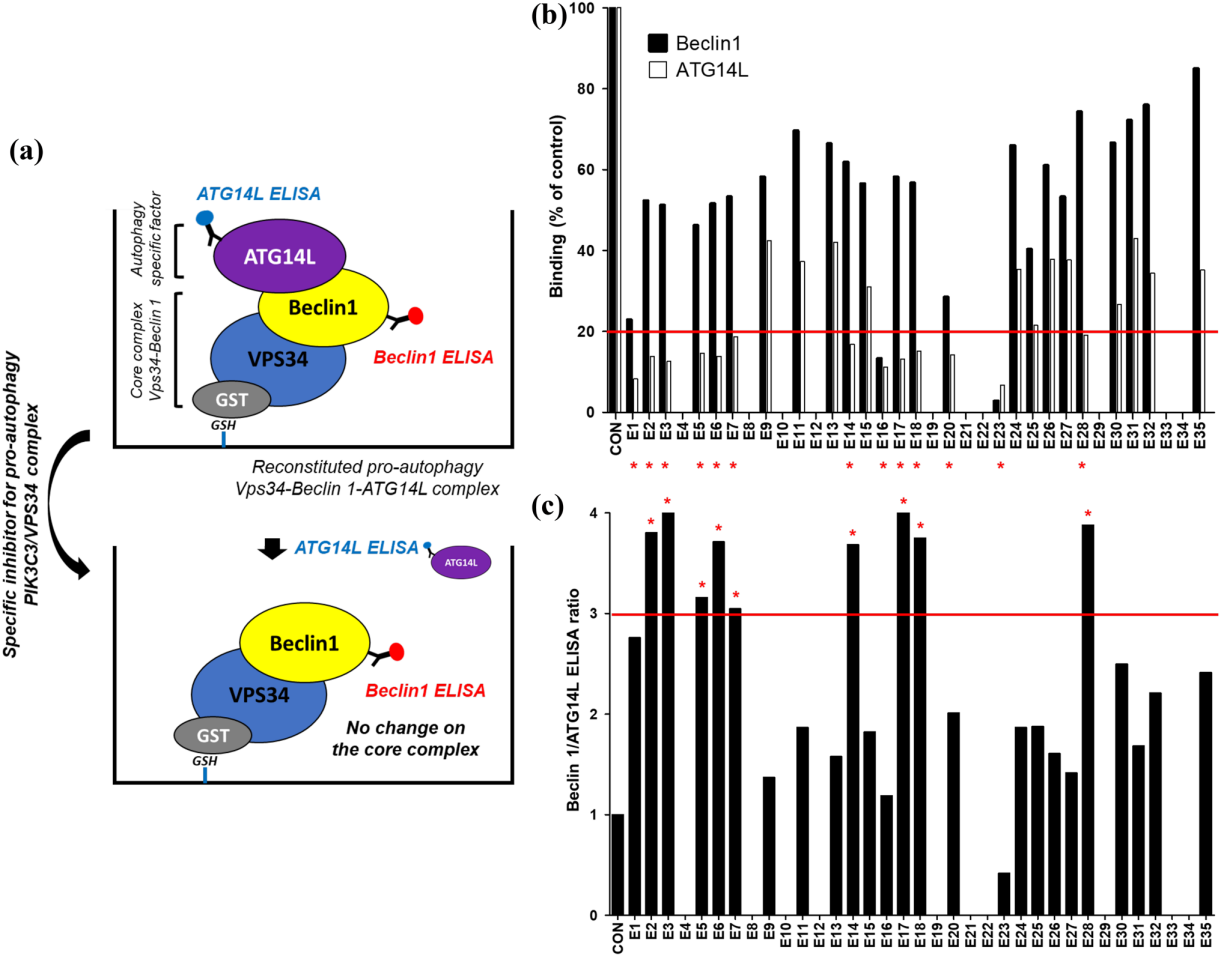
Identification of natural herbal medicinal extracts specifically disrupting ATG14L binding to PIK3C3/VPS34 complex core unit, Vps34-Beclin 1, in vitro. (**a**) Schematic diagram for an in vitro autophagy-specific inhibitor screening. (**b**,**c**) First round screening of in vitro Vps34-Beclin 1-ATG14L formation assay against a small set of natural herbal medicinal extracts. 35 natural herbal medicinal extracts were added at final 0.2 mg/ml concentration (Each extract was originally dissolved in DMSO at 20 mg/ml and is diluted to 1:100 in assay buffer) and ELISA assays for Beclin 1 and ATG14L, respectively, were performed (**b**) and the resulting ELISA signal ratio between Beclin 1 and ATG14L was represented (**c**). The relative amount of ATG14L or Beclin 1 bound to the immobilized Vps34 shown in (**b**) is shown as % of control under 1% DMSO. A red line in (**b**) and (**c**) represents an arbitrary cut-off for the inhibitor efficiency: more than 80% ATG14L binding (**b**) and more than 3 in the ELISA signal ratio between Beclin 1 and ATG14L (**c**). * denotes the candidates that passed the cut-off.

**Figure 2:**
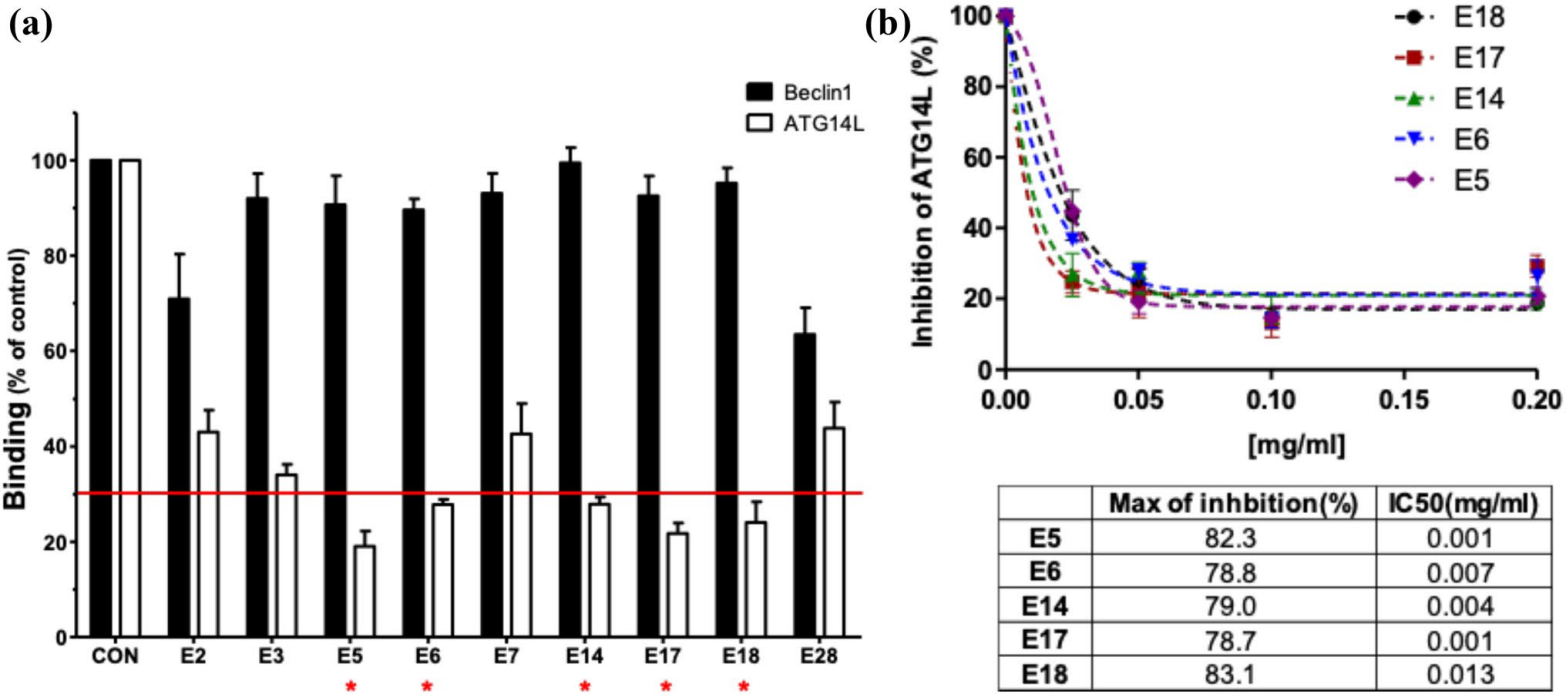
The second round screening and the analysis of inhibition potency of the hit extracts. (**a**) Second in vitro Vps34-Beclin 1-ATG14L complex formation assay. The same experiment shown in Fig. 1B was repeated against nine candidate extracts from the first round screening (E2, E3, E5, E6, E7, E14, E17, E18, and E28) at fourfold diluted condition (0.05 mg/ml). The relative ATG14L or Beclin 1 bindings are represented as % of the control with 0.25% DMSO instead of the extract. * denotes the candidates that passed the cut-off. (**b**) The kinetic analysis for the inhibition of ATG14L binding to the complex core unit, Vps34-Beclin 1. The complex formation assay was carried out against the five extracts that were satisfied with the cut-off on (**a**). The ELISA assays against both ATG14L and Beclin 1 were conducted in a manner dependent of the concentration of the five extracts. The ELISA results were plotted and analyzed their maximum inhibition (%) and IC50 by Prism GraphPad program v5. All data are obtained from the experiments in duplicate.

Although the underlying mechanism is still largely unknown, accumulating reports have shown that the medicinal effects of various natural herbal extracts used in oriental medicine are dependent on autophagy^30^. It makes the natural herbal medicinal extracts as an attractive repertoire for developing autophagy-specific modulators. Especially, considering the complexity of protein–protein binding interface, the natural extract may be a good starting resource to obtain PPI inhibitor to prevent ATG14L from binding to PIK3C3/VPS34 complex core unit, because a single small molecule may not good enough to interfere with PPI. For the inhibitor screening, we have selected 35 natural herbal medicinal extracts (E1-E35, Table 1), which are mostly used as anti-inflammatory, anti-tumor, or anti-bacterial/viral agents in oriental medicine, to make up the screening library. Considering the significance of autophagy in immune responses, these natural herbal extracts are expected to have a role in autophagy. In the inhibitor screening, the purified ATG14L protein was added into the immobilized PIK3C3/VPS34 complex core unit, Vps34-Beclin 1, to reconstitute pro-autophagy complex, Vps34-Beclin 1-ATG14L, in which natural herbal extract was added to examine the complex formation by ELISA against ATG14L and Beclin 1, respectively (Fig. 1a). When the extract specifically interferes with PPI between ATG14L and Beclin 1, ELISA signal for ATG14L will be decreased, but the Beclin 1 signal will not change. Therefore, the ratio of Beclin 1/ATG14L ELISA value will be increased. In the initial screening shown in Fig. 1b, ten natural extracts (E4, E8, E10, E12, E19, E21, E22, E29, E33, and E34) showed neither Beclin 1 nor ATG14L ELISA signals, suggesting that they might affect the complex core unit Vps34-Beclin 1 on the plate at 0.2 mg/ml concentration. Except them, thirteen extracts caused the decrease in ATG14L signal more than 80% (Fig. 1b), of which the nine extracts have changed Beclin 1 signal less than 50% decrease so that their Beclin 1/ATG14L ELISA signal ratio was greater than 3 (Fig. 1c). For these nine candidates from the first round screening, we have repeated the same experiment with them at fourfold diluted condition (0.05 mg/ml) to obtain more potent inhibitors. As shown in Fig. 2a, E5 (*Forsythia koreana* Nakai), E6 (*Artemisia scoparia* Waldst. & Kitam.), E14 (*Morus bombycis* Koidz.), E17 (*Ampelopsis japonica* Makino), and E18 (*Thuja orientalis* L.) still potently inhibited ATG14L binding more than 70%, and they have almost no effect on the complex core unit (less than 10% decrease in Beclin 1). To evaluate the kinetics for the inhibition of ATG14L binding to the complex core unit, Vps34-Beclin 1, the dose-dependent inhibition of ATG14L binding was tested and their inhibition curve was plotted to obtain the maximum inhibition (%) and IC50. As shown in Fig. 2b. All five natural herbal extracts inhibit ATG14L binding at maximum around 80% and their IC50 was in the range from 1 μg/ml (E5) to 13 μg/ml (E18).

**Table 1:** List of 35 natural herbal medicinal extracts. All listed natural herbal extracts were prepared by a standard extraction protocol using either ethanol extraction or steam distillation. They are lyophilized and then dissolved in DMSO at the concentration of 20 mg/ml, which was diluted with the binding assay buffer or cell culture medium in the experiment as indicated.

	Name		Name
E1	Rubus foliolosus (Rubi Fructus)	E19	Zingiber officinale (Zingiberis Rhizoma)
E2	Salviae Miltiorrhizae Radix	E20	Paeomia suffruticosa (Moutan Cortex Radicis)
E3	Cassia tora (Cassiae Semen)	E21	Panax ginseng (Ginseng Radix Alba)
E4	Foeniculum vulgare Gaertner (Foeniculi Fructus)	E22	Coriandrum sativum (Coriandri Herba cum Radix)
E5	Forsythia koreana Nakai (Forsythiae Fructus)	E23	Sanguisorba officinalis (Sanguisorbae Radix)
E6	Artemisia scoparia (Artemisiae Scopariae Herba)	E24	Scutellaria baicalensis (Scutellariae Radix)
E7	Rheum coreanum Nakai (Rhei Rhizoma)	E25	Paeonia lactiflora Pall (Paeoniae Radix)
E8	Morus alba (Mori Folium)	E26	Hedera japonica Tobler (Hedera rhombea)
E9	Angelica gigas Nakai (Angelicae Gigantis Radix)	E27	Citrus unshiu Markovich (Citrii Unshiu Immaturi Pericarpium)
E10	Lycium chinense Mill (Lycii Fructus)	E28	Machilus thunbergia (Magnoliae Cortex)
E11	Rheum officinale Baillon (Rhei Undulati Rhizoma)	E29	Acorus gramineus Sol (Acori Gramineri Rhizoma)
E12	Peucedanum praeruptorum Dunn (Peucedani Radix)	E30	Perilla frutescens var. crispa (Perilla Herba)
E13	Peucedanum japonicum Thunberg (Glehniae Radix cum Rhizoma)	E31	Angelica dahurica (Angelicae Dahuricae Radix)
E14	Morus bombycis Koidz (Mori Fructus)	E32	Chrysanthemum indicum (Chrysanthemi Flos)
E15	Cyperus rotundus (Cyperi Rhizoma)	E33	Houttuynia cordata (Houttuyniae Herba)
E16	Saururus chinensis Baill (Houttuyniae Herba)	E34	Cirsium japonicum var. ussuriense (Cirsii Herba)
E17	Ampelopsis japonica Makino (Ampelopsis Radix)	E35	Prunella vulgaris var. lilacina NAKAI (Prunellae Spica)
E18	Thuja orientalis (Biotae Folium)

### An alcoholic extract of *T. orientalis* L. inhibits the starvation-induced autophagy in cells

**Figure 3:**
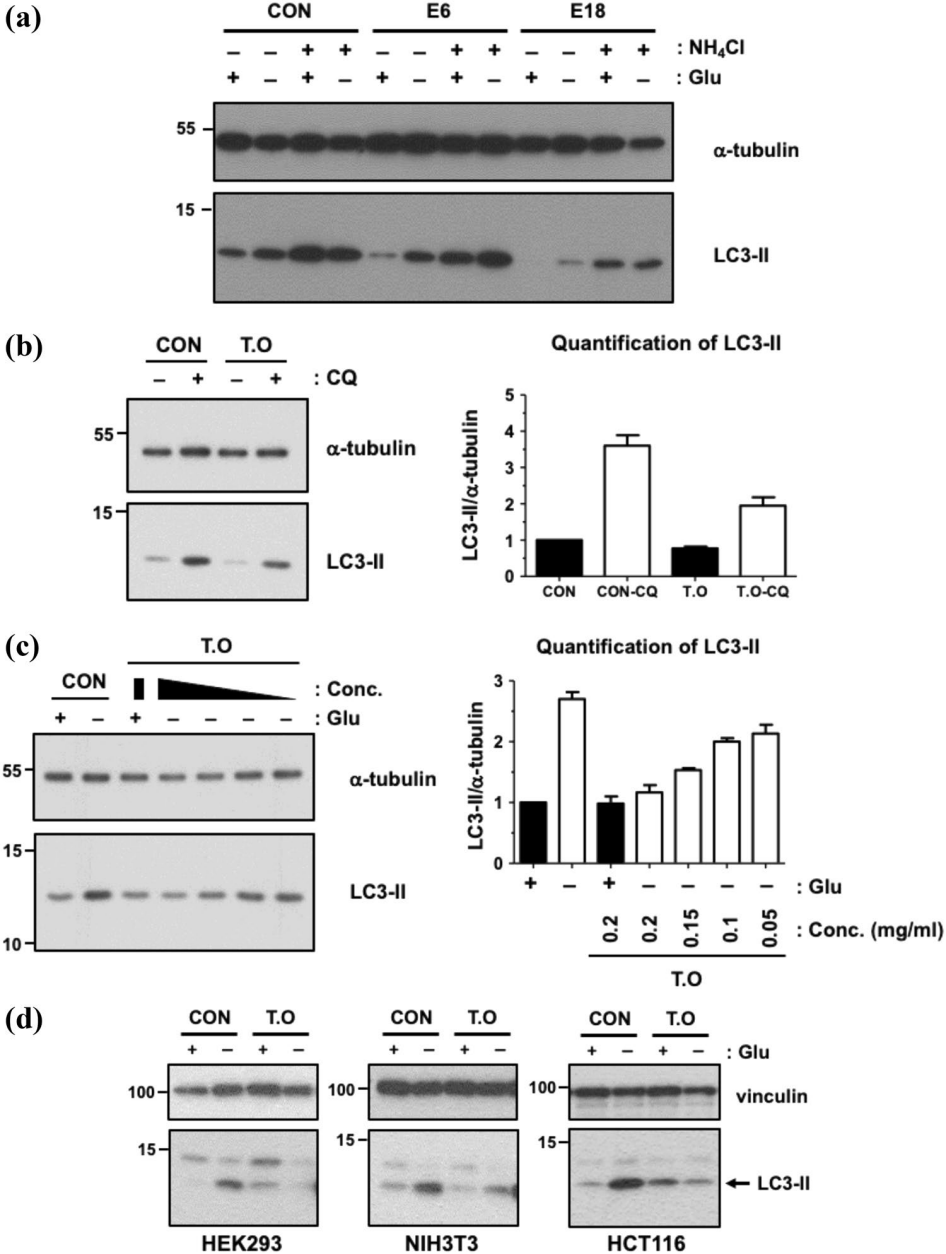
*T. orientalis* inhibits the starvation-induced autophagy. (**a**) E18 (*Thuja orientalis*) inhibits the starvation-induced autophagy. MEFs were starved for glucose (Glu) overnight in the presence of 0.2 mg/ml of the extracts, in which 10 mM NH_4_Cl was added as indicated. (**b**) Autophagy flux was decreased, but not blocked, by *T. orientalis*. MEFs were grown in the DMEM culture medium with or without 10 μM Chloroquine (CQ) for 8 h to block lysosome-dependent LC3-II degradation, thereby leading to LC3-II accumulation. To test the effect of *T. orientalis* (T.O) on the basal autophagy flux, 0.2 mg/ml of T.O was added to examine autophagy as evidenced by LC3-II immunoblot. (**c**) Dose-dependent inhibition of the starvation-induced autophagy by *T. orientalis*. MEFs were starved for glucose for 8 h, in which the decreasing amount of T.O (0.2, 0.15, 0.1, and 0.05 mg/ml) was added as indicated. (**d**) The starvation-induced autophagy was inhibited by *T. orientalis* in various cell lines. HEK293, NIH3T3, and HCT116 were starved for glucose overnight in the presence or absence of 0.2 mg/ml *T. orientalis* as indicated. Quantification of LC3-II level with respect to the level of α-tubulin was obtained by analyzing the intensities of LC3-II and α-tubulin on immunoblots from 3–5 independent experiments, of which one representative image was shown. Image analysis was performed by Image J program. The original unprocessed full-length images of the western blots in this study can be found in Supplemental Fig. 1.

**Figure 4:**
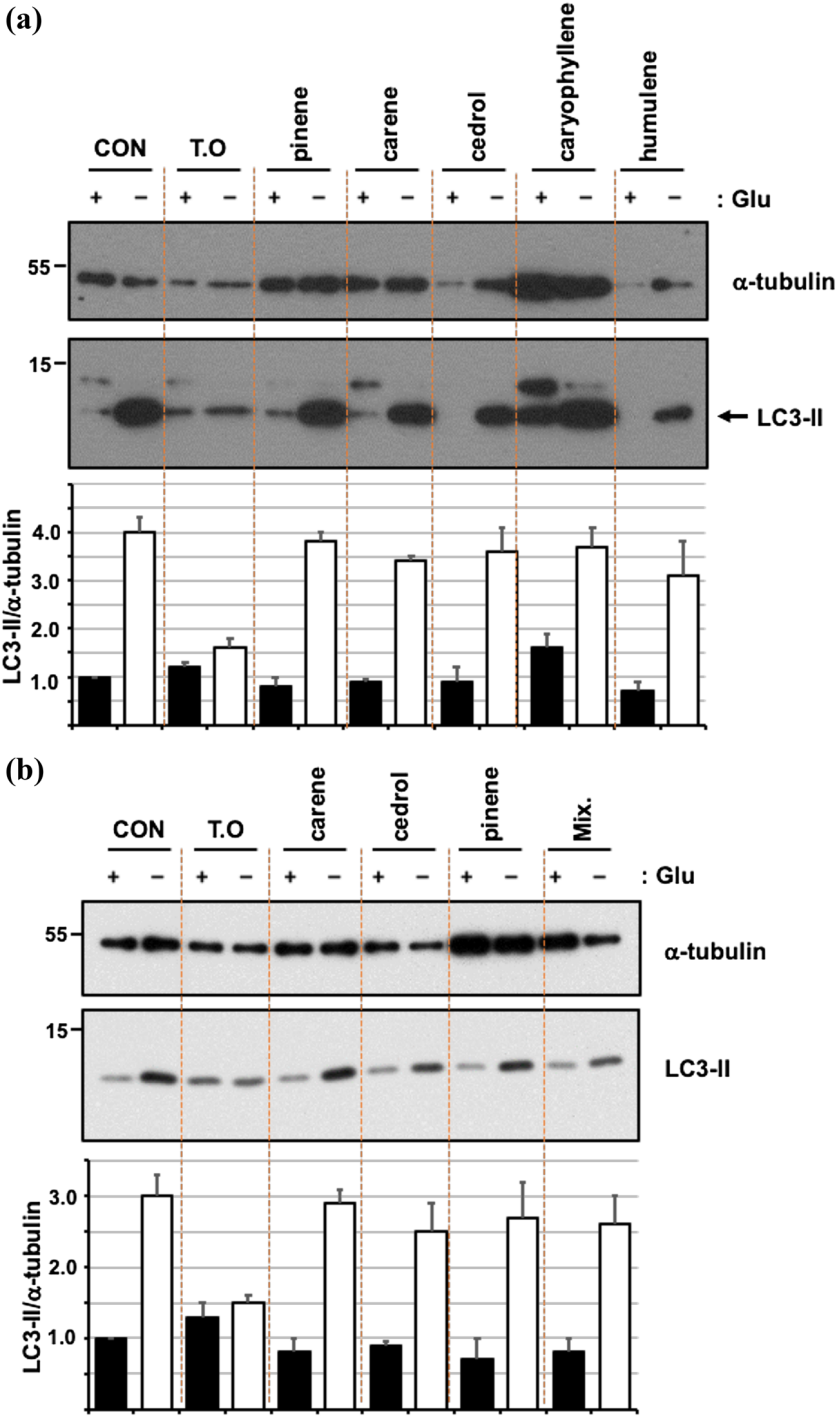
Evaluation of major components in *T. orientalis* (T.O) for the inhibition of the starvation-induced autophagy. (**a**) MEFs were starved for glucose (Glu) overnight, in which five major components (more than 5% in the extract total) have treated at the following concentrations: α-pinene, 123 μM; Δ-3-carene, 392.5 μM; α-cedrol, 112.3 μM; β-caryophyllene 1.1 mM; and α-humulene, 169.9 μM. (**b**) The same experiment was repeated against carene, cedrol, pinene, and the mixture of these three components (Mix.) under 8 h starvation condition. Autophagy and the protein loading in the experiments were examined by immunoblots against LC3-II and α-tubulin, respectively. A representative image from 3–5 different immunoblots and their quantification of LC3-II level/α-tubulin were shown below.

Next, we have determined the effect of these five candidate extracts on cellular autophagy responses. The nutrient starvation is long-believed to induce autophagy to maintain cellular energy homeostasis ^2^. The cells were starved with glucose, a primary energy source in cell culture medium, in which each extract was added. For overnight starvation, almost of all cells were dead in the presence of E5, E14, or E17 at the concentration of 0.2 mg/ml, suggesting their high toxicity in this experimental condition. In contrast, cell viability was little affected by either E6 or E18. As shown in Fig. 3a, glucose starvation induced autophagy as evidenced by the immunoblot showing the increase in LC3-II upon the starvation. In this condition, E6 has no effect on the starvation-induced autophagy, but E18 dramatically decreased the starvation-induced LC3-II, demonstrating that E18, an alcoholic extract of *Thuja orientalis* L. leaves (hereafter referred to as *T. orientalis*), functions as an inhibitor of autophagy in response to the starvation. Notably, under *T. orientalis*, both LC3-II in the absence or presence of a lysosome blocker, NH_4_Cl, was much lower than the control. NH_4_Cl suppresses autophagy flux to block LC3-II degradation in autolysosome, thereby accumulating LC3-II, but it was significantly suppressed by *T. orientalis*. Similarly, the inhibition of the basal autophagy flux by another lysosomal inhibitor, Chloroquine (CQ), was also significantly decreased by *T. orientalis* (Fig. 3b). These results collectively suggest that *T. orientalis* functions as an autophagy inhibitor in a manner different to the lysosome blockers and its inhibition may occur before lysosomal degradation, probably, at the initiation step regulated by PIK3C3/VPS34 complex as it was discovered as an inhibitor of PIK3C3/VPS34 complex formation in vitro. Therefore, *T. orientalis* is expected to decrease autophagy flux, but not block the flux. Inhibition of *T. orientalis* on the starvation-induced autophagy was increased in a dose-dependent manner (Fig. 3c). Also, the inhibition of the starvation-induced autophagy was observed in several other cell lines, such as HEK293, NIH3T3, and human cancer cell line, HCT116 (Fig. 3d), suggesting that it may function as a general autophagy inhibitor at least upon the nutrient starvation. Next, we have tried to identify an active single component in *T. orientalis* required for the autophagy inhibition. In search of the literature analyzing the components of *T. orientalis*^31,32^, we have found that it was made up of more than 20 components, of which top five components more than 5% in the extract total were chosen^31^: α-pinene (~ 29%), Δ-3-carene (~ 20%), α-cedrol (~ 10%), β-caryophyllene (~ 8%), and α-humulene (~ 6%). We have treated these single components at the maximum concentration at which the cells were viable at least more than 50% in overnight treatment and examined their inhibition potency against the starvation-induced autophagy (Fig. 4a). Unfortunately, under the overnight starvation, although there were variations in cell viability, none of any single component was as effective as *T. orientalis* in the inhibition of the starvation-induced autophagy. In addition, top three components (pinene, carene, and cedrol) were mixed together to test their efficacy of autophagy inhibition, this mixture barely inhibited the starvation-induced autophagy (Fig. 4b). It suggests that inhibition effect of *T. orientalis* on the starvation-induced autophagy may rely on either the other component present in the smaller amount in the extract or the more complex combination of the extract components.

### *T. orientalis* specifically decreases pro-autophagy PIK3C3/VPS34 complex containing ATG14L (or UVRAG) in cells

**Figure 5:**
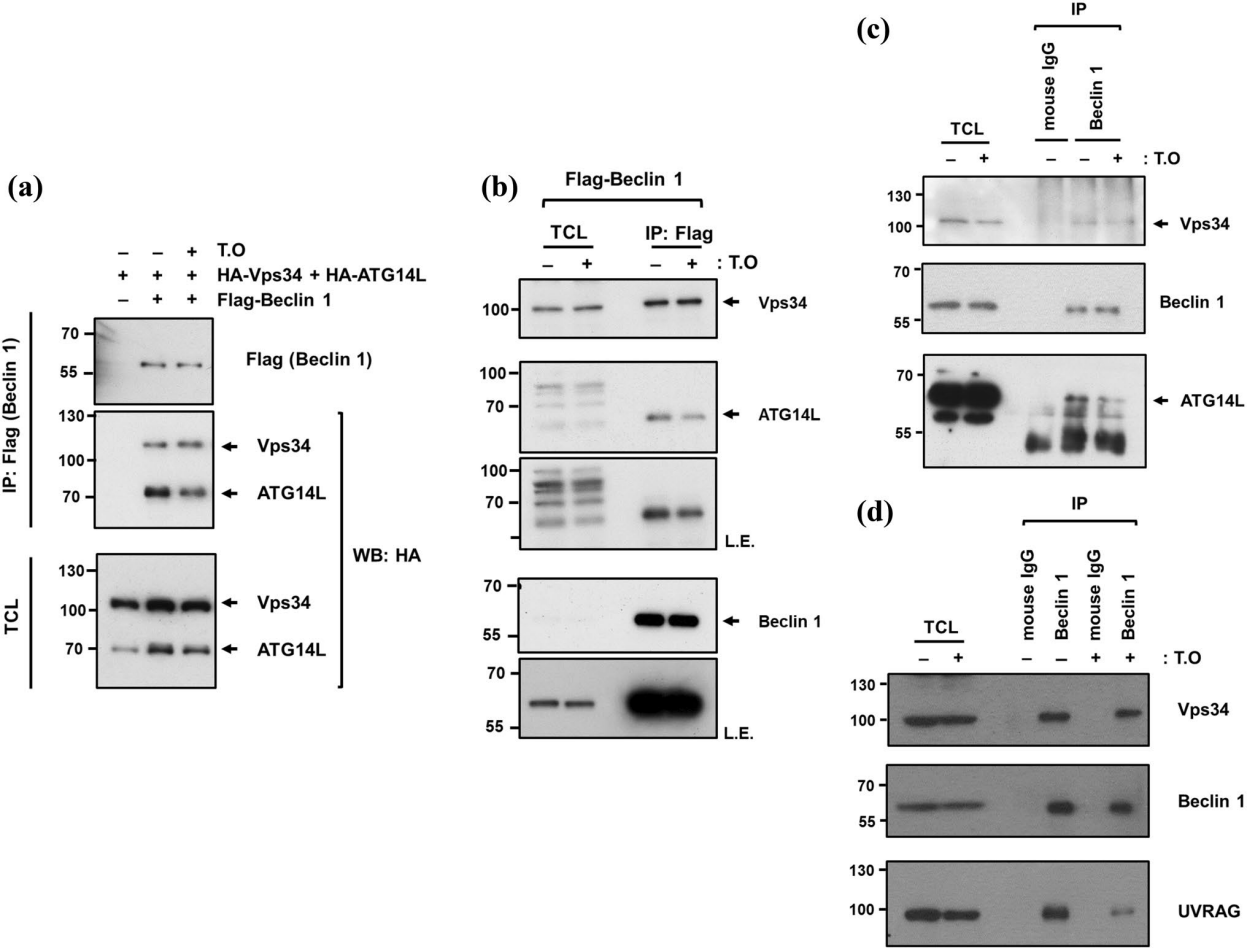
*T. orientalis* (T.O) specifically disrupt the binding of autophagy-specific protein ATG14L (or UVRAG) to the PIK3C3/VPS34 complex core unit, Vps34-Beclin 1, but not affecting the interaction between Vps34 and Beclin 1. (**a**) Vps34-Beclin 1-ATG14L complex was immunoprecipitated from the transfected HEK293 cells as indicated. Before immunoprecipitation, cells were incubated with or without 0.2 mg/ml of *T. orientalis* for 2 h. The complex was immunoprecipitated by anti-Flag antibody targeting Flag-Beclin 1 and Co-IPs of HA-Vps34 and HA-ATG14L were determined by immunoblots using anti-HA antibody. (**b**) Co-immunoprecipitations of the endogenous Vps34 and ATG14L by the ectopic Flag-Beclin 1 in the transfected HEK293 cells. After 2 day transfection of Flag-Beclin 1, the cells were treated with 0.2 mg/ml of *T. orientalis* for 2 h and then, Flag-Beclin 1 was immunoprecipitated. Co-IPs of both endogenous Vps34 and ATG14L were examined by their immunoblots. (**c**,**d**) Immunoprecipitation of the endogenous Vps34-Beclin 1-ATG14L complex from MEFs (**c**) and Vps34-Beclin 1-UVRAG from HEK293 (**d**), respectively. The cells were incubated with or without 0.2 mg/ml of *T. orientalis* for 4 h and then, the indicated pro-autophagy PIK3C3/VPS34 complex were determined by immunoblots against the endogenous Vps34, Beclin 1, ATG14L, or UVRAG as indicated. Mouse IgG was used for the negative IP control in (C-D). Total cell lysates (TCL) were also examined to determine the protein amounts of Vps34, Beclin 1, ATG14L, or UVRAG used in the immunoprecipitation.

Finally, to examine inhibition mechanism of *T. orientalis* on autophagy, we have analyzed pro-autophagy PIK3C3/VPS34 complex in cells because it was originally identified as an inhibitor targeting the formation of pro-autophagy complex, Vps34-Beclin 1-ATG14L, in vitro. To this end, the plasmids encoding HA-Vps34, Flag-Beclin 1, and HA-ATG14L, respectively, were transfected to HEK293 cells and Flag-Beclin 1 was immunoprecipitated. Co-immunoprecipitations (Co-IPs) of HA-Vps34 and HA-ATG14L were tested for their binding to Beclin 1 by their immunoblots. As shown in Fig. 5a, both HA-Vps34 and HA-ATG14L were co-immunoprecipitated by Flag-Beclin1 in control, indicating the formation of pro-autophagy Vps34-Beclin 1-ATG14L complex in this experimental setting. However, in the presence of *T. orientalis*, Co-IP of ATG14L was significantly decreased in the Beclin 1 immune-complex, whereas Co-IP of Vps34 was not affected. To further confirm that *T. orientalis* specifically inhibits Beclin 1-ATG14L binding, we carried out two different endogenous Vps34 and ATG14L Co-IP assay. First, we have transfected Flag-Beclin1 into HEK293 cells and then Co-IPs of the endogenous Vps34 and ATG14L in Flag-Beclin 1 immune-complex was examined by immunoblots (Fig. 5b). Consistent with the transfection result in Fig. 5a, almost the same amount of the endogenous Vps34 was co-immunoprecipitated regardless of *T. orientalis*, but Co-IP of ATG14L was significantly decreased in *T. orientalis* treatment condition. Second, we have conducted Co-IP assay targeting the endogenous Beclin 1 in both MEFs and HEK293. Consistently, we obtained the same Co-IP results for the endogenous Vps34 and ATG14L in the endogenous Beclin 1 immune-complex from MEFs (Fig. 5c), in which only Co-IP of the endogenous ATG14L was decreased by *T. orientalis*. We did not obtain a reliable Co-IP result for ATG14L in the endogenous Beclin 1 immune-complex from HEK293, but we have obtained very clear Co-IP of the endogenous UVRAG in this cell (Fig. 5d). Like as ATG14L, Co-IP of the endogenous UVRAG was dramatically decreased upon *T. orientalis*, but Co-IP of the endogenous Vps34 was not. Considering that ATG14L and UVRAG compete with Beclin 1 binding and their binding regions on Beclin 1 are closely overlapped, it is expected that *T. orientalis* can disrupt both ATG14L and UVRAG containing PIK3C3/VPS34 complex. These results collectively support that *T. orientalis* specifically target pro-autophagy PIK3C3/VPS34 complex containing either ATG14L or UVRAG by disrupting these autophagy-specific subunit binding to Beclin 1, but not the interaction of the complex core unit, Vps34-Beclin 1, as proposed in in vitro complex formation screening.

## Discussion

In this study, we have identified an alcoholic extract of *T. orientalis* L. leaves as an autophagy-specific inhibitor by targeting pro-autophagy PIK3C3/VPS34 complex containing either ATG14L or UVRAG. We have screened a customized natural product library consisting of 35 herbal medicinal extracts with anti-inflammation, anti-tumor, or anti-bacterial/viral activities, which are closely related to pathophysiological roles of autophagy. An in vitro pro-autophagy Vps34-Beclin 1-ATG14L complex formation assay was carried out in the presence of each natural herbal extract and then, the selected hit extracts were tested their autophagy inhibition activities in cells. Among 35 extracts, *T. orientalis* successfully inhibited the starvation-induced autophagy and specifically disrupt pro-autophagy PIK3C3/VPS34 complex formation in both in vitro and cells. It specifically targets Beclin 1-ATG14 (or UVRAG) binding, but not the complex core unit, Vps34-Beclin 1, binding. These results indicate that *T. orientalis* could be an autophagy inhibitor specifically disrupting the pro-autophagy PIK3C3/VPS34 complex by preventing autophagy-specific factors, ATG14L or UVRAG, from the binding to the complex core unit, Vps34-Beclin 1. It is important because the complex core unit Vps34-Beclin 1 is a backbone of the complex, in which Beclin 1 recruits many different subunits as a scaffold subunit to form various PIK3C3/VPS34 complexes in a wide spectrum of cell physiology, such as vesicle trafficking, endocytosis, and amino acid signaling^23^. Therefore, a targeting a pro-autophagy PIK3C3/VPS34 complex by *T. orientalis* may result in the regulation of one specific function of various PIK3C3/VPS34 complexes, autophagy, but not the other functions. In this sense, it is different to other PIK3C3/VPS34 inhibitors, such as 3-MA and Spautin-1, both of which target the complex core unit. 3-MA is a catalytic inhibitor of Vps34^11^ and Spautin-1 targets Beclin 1 degradation^21^, thereby they may affect all PIK3C3/VPS34 complex family members. It should be noted that *T. orientalis* disrupts both ATG14L- and UVRAG-containing pro-autophagy PIK3C3/VPS34 complex. ATG14L and UVRAG participate in the complex by binding to Beclin 1 in a mutually exclusive manner, suggesting that their binding sites on Beclin 1 are overlapped. Therefore, although *T. orientalis* was initially identified to disrupt Beclin 1-ATG14L binding by an in vitro Vps34-Beclin 1-ATG14L complex formation assay, it is expected that it also affects the UVRAG-containing complex. Indeed, it was observed that *T. orientalis* diminished both ATG14L- and UVRAG-containing PIK3C3/VPS34 complex in MEFs or HEK293 cells to inhibit autophagy. Importantly, UVRAG-containing PIK3C3/VPS34 complex is known to play an important role in the maturation of autophagosome as well as endosomal trafficking, whereas ATG14L-containing one is believed to mainly function in the initiation of autophagosome formation^33,34^. Therefore, the fact that *T. orientalis* also disrupt Vps34-Beclin 1-UVRAG complex may result in its undesirable effects in endolysosomal pathway. Notably, the treatment of *T. orientalis* appears to slightly decrease the protein level of ATG14L (Fig. 5a,b), therefore, it is possible that the reduction of Vps34-Beclin 1-ATG14L complex in this condition may result from the decrease in ATG14L protein level, but not the inhibition of Beclin 1-ATG14L binding. However, it is also possible that free ATG14L that do not participate in the complex by *T. orientalis* is unstable to be degraded, decreasing the protein level. One more caution to use *T. orientalis* as an autophagy inhibitor is that it may not work in PIK3C3/VPS34 independent (or bypass) autophagy. Also, if there is any mutation in Beclin1-ATG14L (or UVRAG) binding interface or surrounding regions, this protein–protein interaction inhibitor may be no longer functional in the pro-autophagy PIK3C3/VPS34 complex containing them, thereby no effect on autophagy. Unfortunately, we could not assign a single active component in the extract of *T. orientalis* for autophagy inhibition. It may cause some trouble in developing it as a drug, but the extract of *T. orientalis* has been long and extensively used in the oriental medicine, therefore, it will provide an important clue to develop an autophagy-specific inhibitor as a therapeutic reagent for human diseases.

## Methods

### Materials

Antibodies used in this study are as follows: anti-Vps34 (Cell signaling, #4263), anti-Beclin1 (Cell signaling, #3738; Santa Cruz, sc-48341 for immunoprecipitation), anti-ATG14L (MBL, PD016), anti-UVRAG (Cell signaling, #13115), LC3B (Cell signaling, #2775), α-tubulin (Santa Cruz, sc-23948), anti-vinculin (SIGMA, V9131) anti-Flag HRP conjugate (Sigma, A8592), anti-HA HRP conjugate (Cell signaling, #2999S) and the secondary antibodies conjugated with HRP, anti-Rabbit IgG-HRP (Bethyl, #A120-101P) and anti-Mouse IgG-HRP (Bethyl, #A90-116P). The affinity resins for the protein purification or immunoprecipitation were obtained from SIGMA (Flag-M2 agarose, A2426), GE Healthcare (GSH-Sepharose, 17-0756-01; Protein G-Sepharose, 17-0618-01), and Invitrogen (Protein A-Sepharose, #101041). Chloroquine (C6628), α-pinene (147524), Δ-3-carene (115576), α-cedrol (93483), β-caryophyllene (22075), and α-humulene (5375) were obtained from Sigma-Aldrich. High glucose (25 mM) DMEM (LM-001-07) and glucose-free DMEM (LM001-56) were from Welgene.

### In vitro Vps34-Beclin 1-ATG14L complex formation assay for natural herbal extract library screening

A detailed protocol to prepare the in vitro Vps34-Beclin 1-ATG14L complex formation assay in high-throughput format can be found in our previous reports^29^. Briefly, N-terminally tagged GST-Vps34, Flag-Beclin 1, and Flag-ATG14L were prepared from the transfected HEK293 cells. To reconstitute Vps34-Beclin 1-ATG14L pro-autophagy complex on the microwell plate, purified GST-Vps34 protein was firstly added and bound to glutathione (GSH)-coated 96 well plate and then the Flag-Beclin1 protein and Flag-ATG14L were sequentially added to reconstitute the complex with a defined orientation. Unbound proteins were extensively washed with TBS (10 mM Tris, pH8.0, and 150 mM NaCl) containing 0.5% Tween-20 (TBS-T5). For library screening, each natural herbal medicinal extract was diluted in PBS as indicated, and added to the microwell containing the Vps34-Beclin 1-ATG14L complex in 100 μl volume. After 30 min incubation at room temperature, the extracts were removed and the microwells were extensively washed with TBS-T5. The remaining Flag-Beclin 1 and Flag-ATG14L protein was determined by ELISA against anti-Beclin 1 antibody (1:100 dilution in TBS containing 0.05% Tween-20, TBST) and anti-ATG14L antibody (1:500 in TBST), respectively.

### Cell culture and transfection

HEK293 (ATCC, CRL-1573), NIH3T3 (Korean Cell Line Bank, #21,658), and mouse embryonic fibroblasts (MEFs, a gift from Dr. Kun-Liang Guan, Dept. of Pharmacology, Univ. of California San Diego, La Jolla, CA, USA) cells were cultured in high glucose (25 mM) DMEM medium containing 10% fetal bovine serum (FBS; Invitrogen) and 50 μg/ml penicillin/streptomycin. For HCT116 human colon cancer cell line (ATCC, CCL-247), cells were grown in RPMI-1640 (Welgene) containing the same serum and antibiotics mixture. To express pro-autophagy PIK3C3/VPS34 complex in cells, the mammalian expression plasmids encoding Vps34, Beclin 1, and ATG14L, respectively, with appropriate N-terminal tags were transfected into HEK293 cells by polyethylenimine (PEI, SIGMA) method as previously reported^35^. After 2 day incubation, the transfected HEK293 cells were washed with ice-cold PBS and lysed in Mild Lysis Buffer (MLB, 10 mM Tris, pH7.5, 2 mM EDTA, 100 mM NaCl, 1% NP-40, 50 mM NaF, 1 mM Na3VO4, and protease inhibitor cocktail) to harvest the total cell lysates for protein purification, immunoblots and immunoprecipitation. Also, the total cell lysates for endogenous protein analysis was also obtained with MLB as above.

### Preparation of alcoholic extracts of *T. orientalis* L. leaves

The commercially available air-dried *T. orientalis* L. leaves were purchased from the Korean traditional medicine market (Seoul Yangnyeong market). A detailed preparation procedure is demonstrated in elsewhere^32^. Briefly, the dried leaves of *T. orientalis* L. have been extracted in 70% ethanol with stirring for 24 h at room temperature. The resulting alcoholic extract was filtrated, concentrated in a rotary vacuum evaporator under the heating at 50 °C, and finally lyophilized to the powder. The powder was dissolved in DMSO and kept on − 20 °C.

### Immunoblotting and immunoprecipitation

Protein samples were resolved on SDS/PAGE and then, electrophoretically transferred onto a PVDF membrane. The membranes were blocked with TBST buffer containing 5% skim milk at room temperature for 1 h. The membranes were incubated with a primary antibody against the indicated proteins, which is diluted in TBST buffer containing 1% BSA to 1:1000 at 4 °C overnight, followed by the reaction with HRP-conjugated secondary antibodies at room temperature for additional 1 h. Antibody detection was carried out using ECL detection kit (PIERCE). For immunoprecipitation, 0.5–1 mg of total cell lysates reacts with the antibody-coupled bead complex, which was prepared by incubation of a specific target antibody with protein A (or G)-coupled Agarose in TBST containing 5% BSA at 4 °C overnight. The immunoprecipitation reaction was carried out at 4 °C for 3–5 h with a gentle rocking. Unbound proteins were removed by an extensive washing with MLB and the immune-complex was analyzed by immunoblots against the antibody recognizing the protein of interest.

## Supplementary Information

Supplementary Figures.
