# Supplementary material for: An alcoholic extract of Thuja orientalis L. leaves inhibits autophagy by specifically targeting pro-autophagy PIK3C3/VPS34 complex
Source: Sci Rep. 2021 Sep 6;11:17712. doi: 10.1038/s41598-021-97216-4 (PMC8421415; doi:10.1038/s41598-021-97216-4)

The original full-length images for western blots in Figure 3A

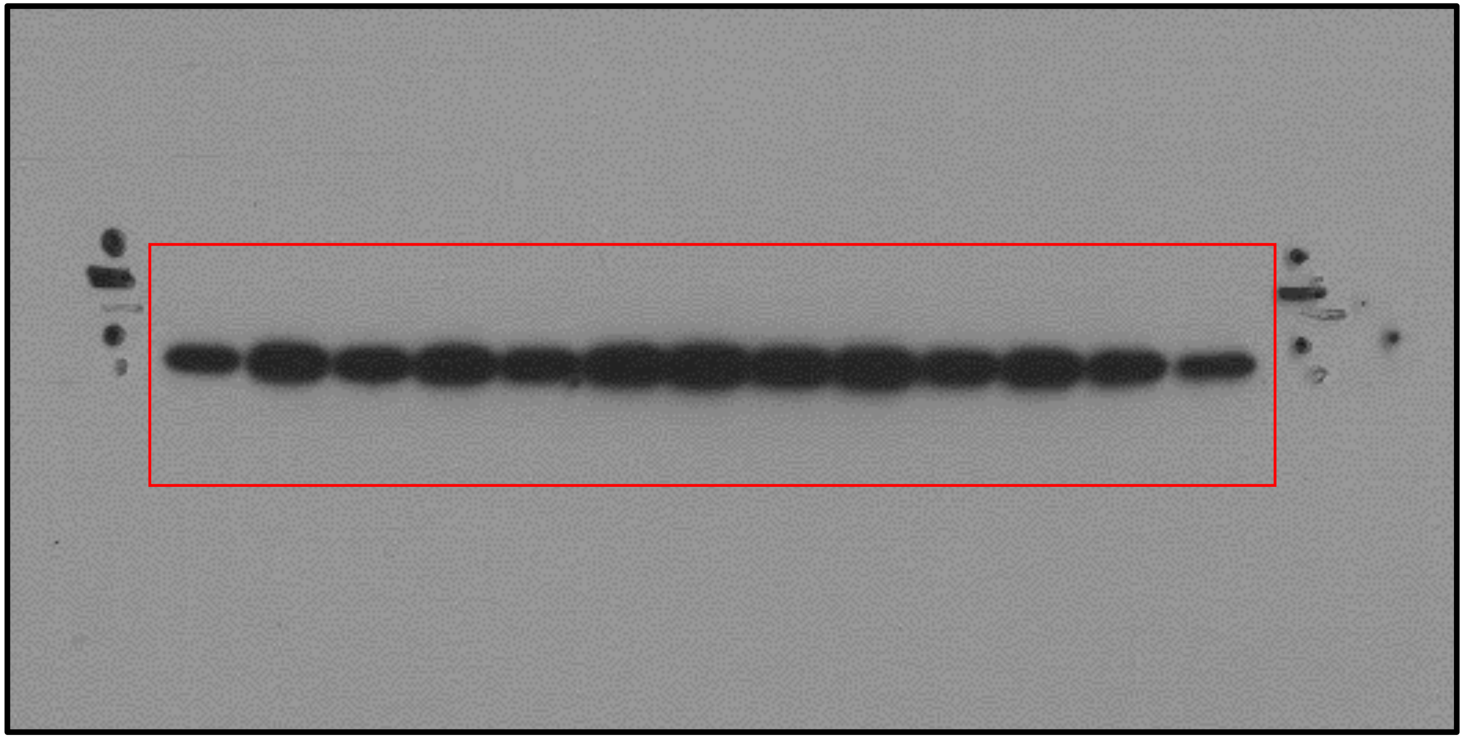

α-Tubulin

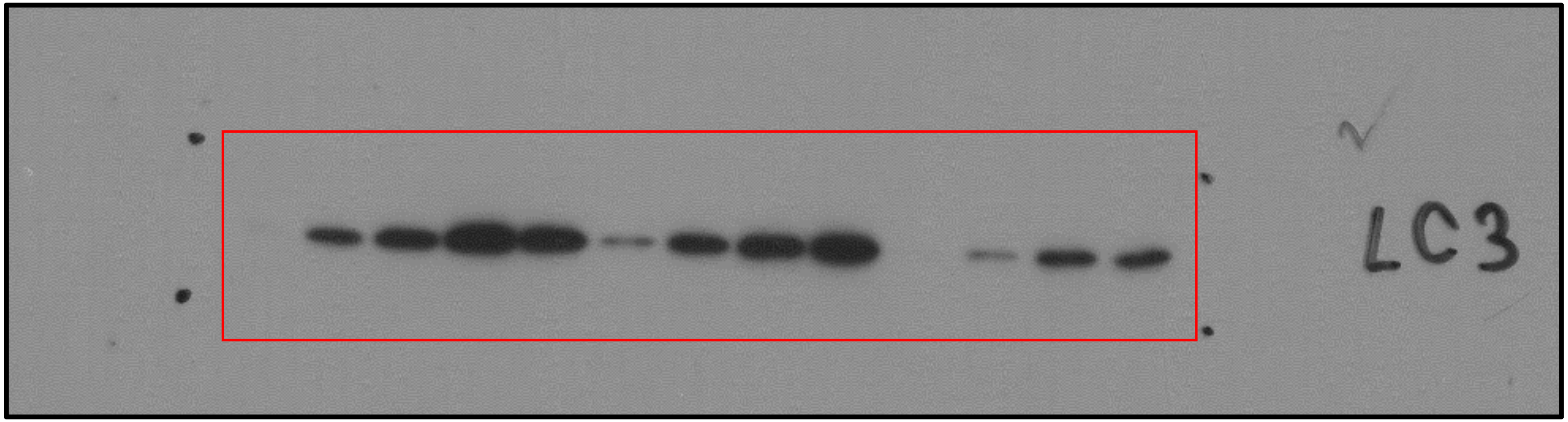

LC3-II

The original full-length images for western blots in Figure 3B

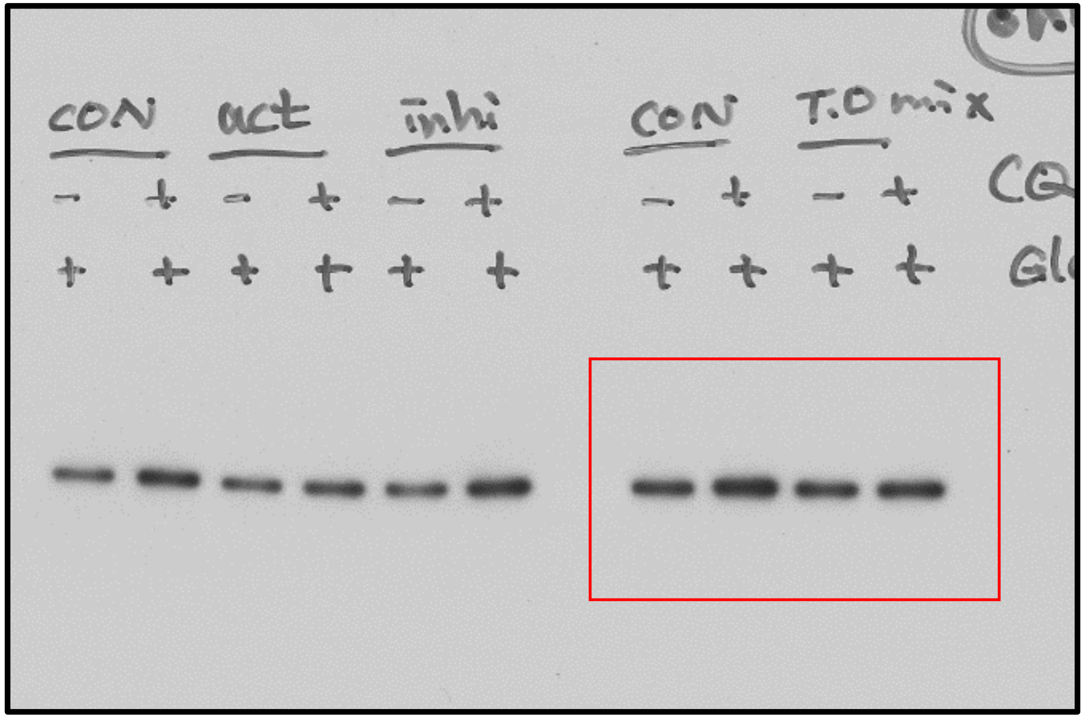

α-tubulin

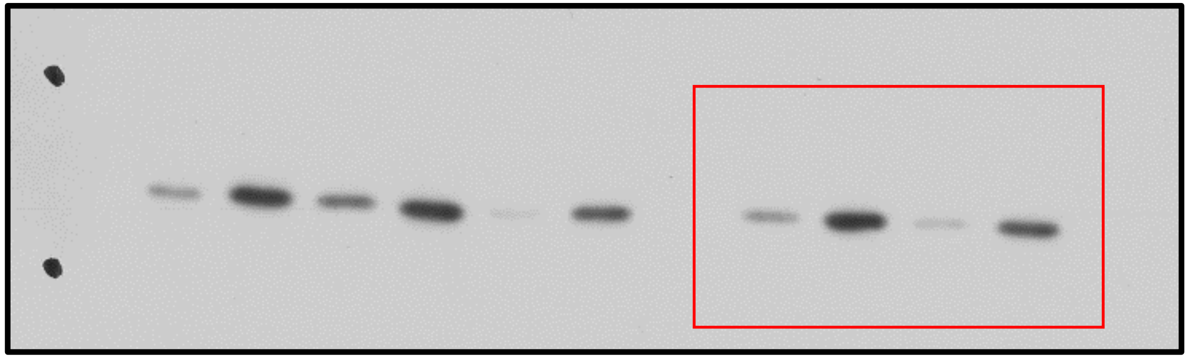

LC3-II

The original full-length images for western blots in Figure 3C

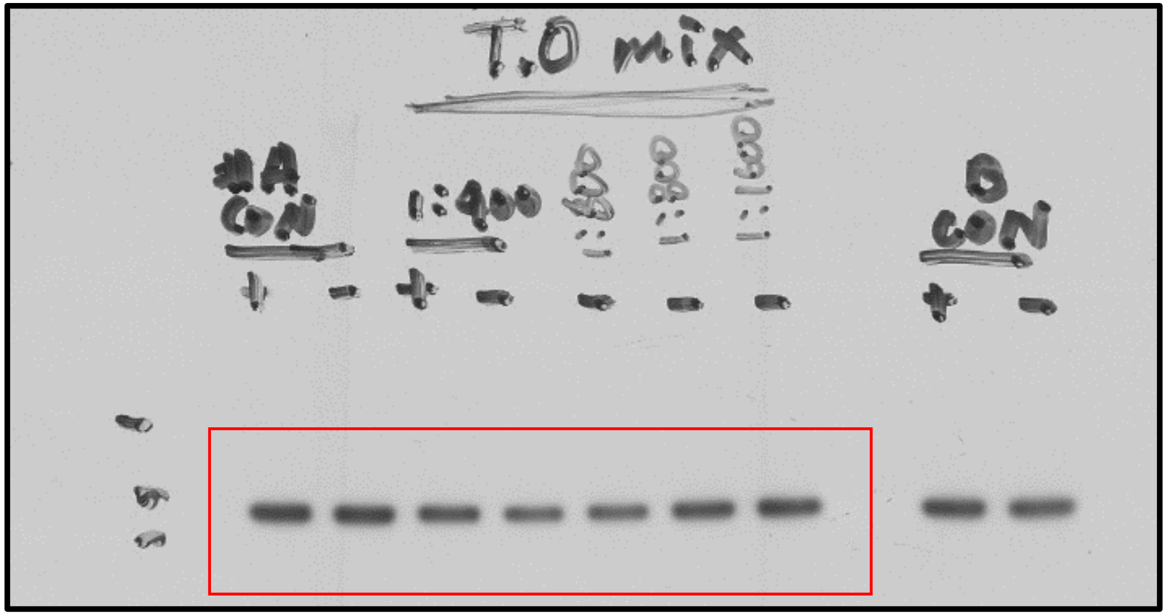

α-tubulin

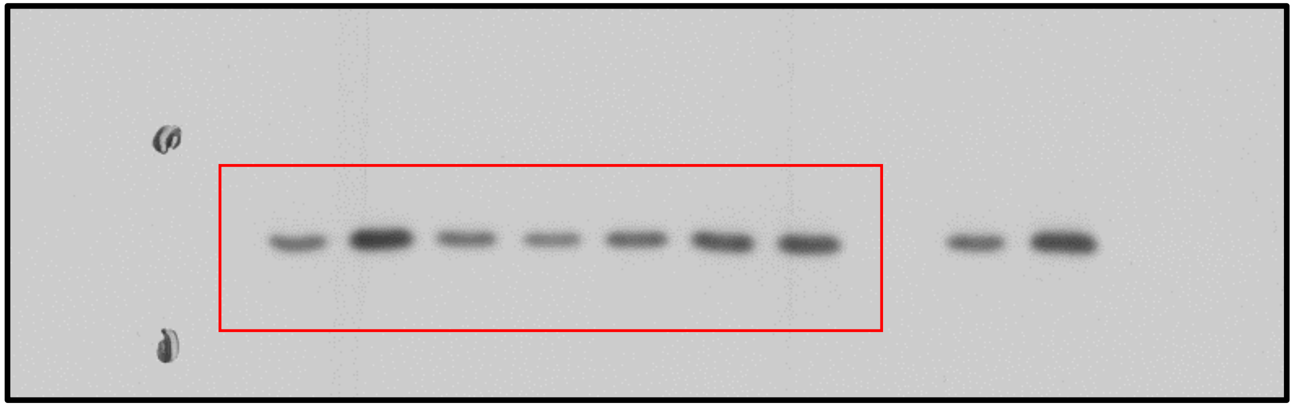

LC3-II

The original full-length images for western blots in Figure 3D

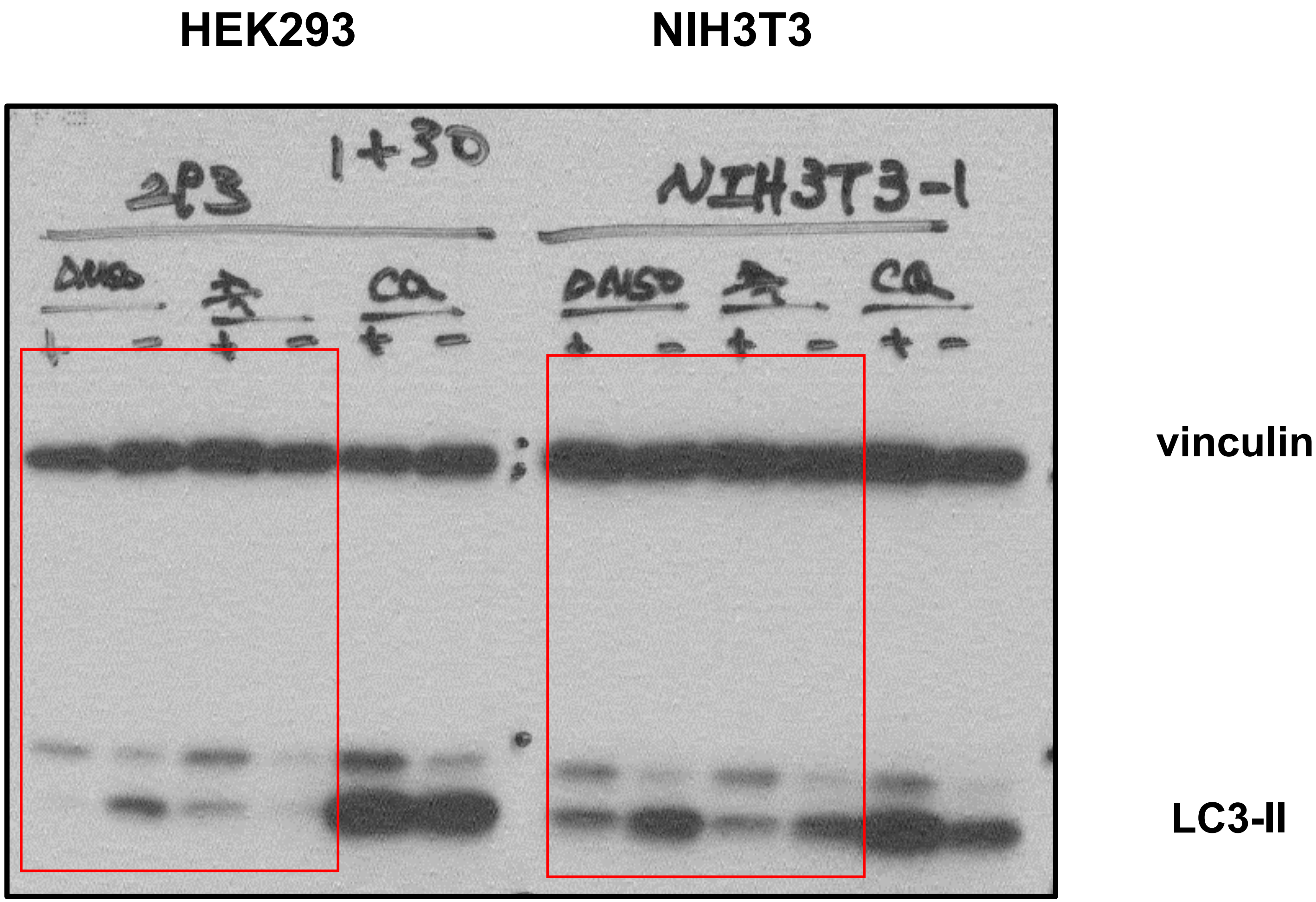

The original full-length images for western blots in Figure 3D

HCT116

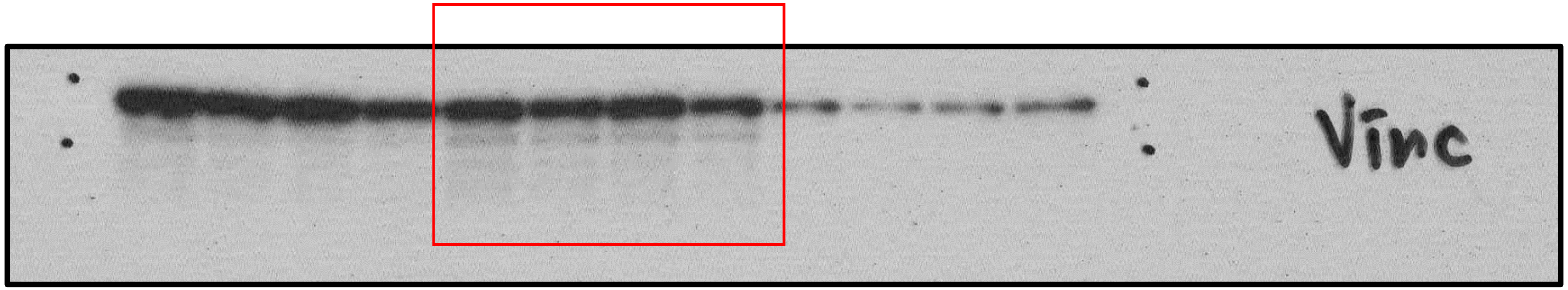

vinculin

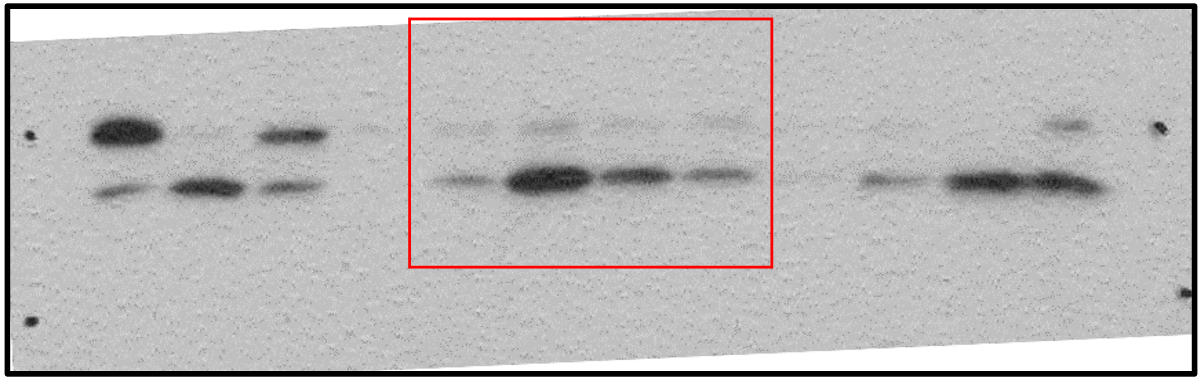

LC3-II

The original full-length images for western blots in Figure 4A

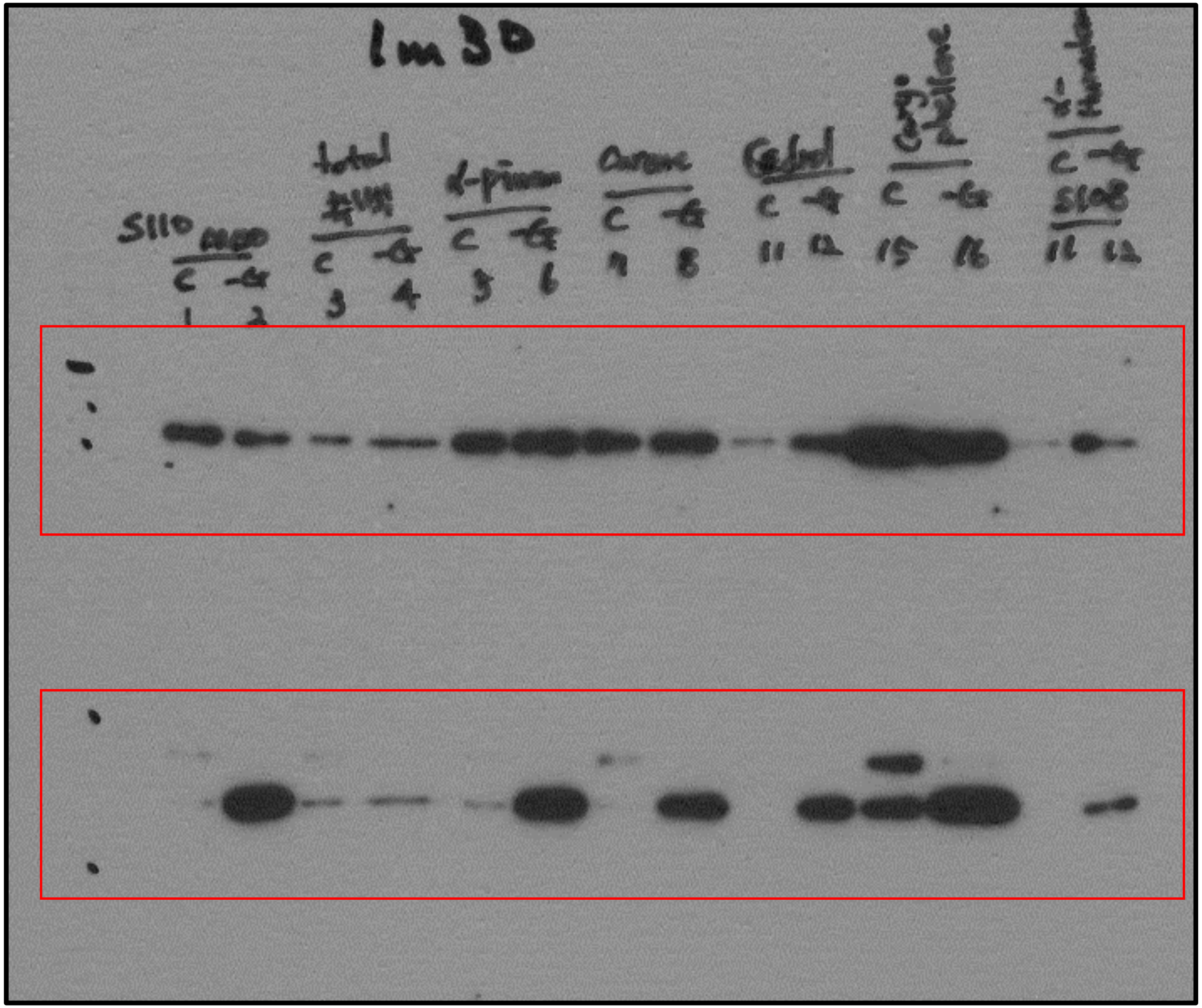

α-tubulin

LC3-II

The original full-length images for western blots in Figure 4B

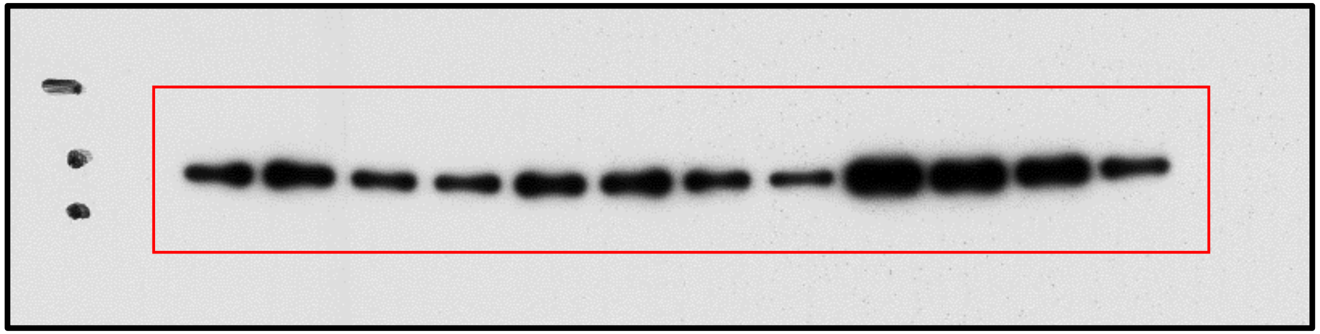

α-tubulin

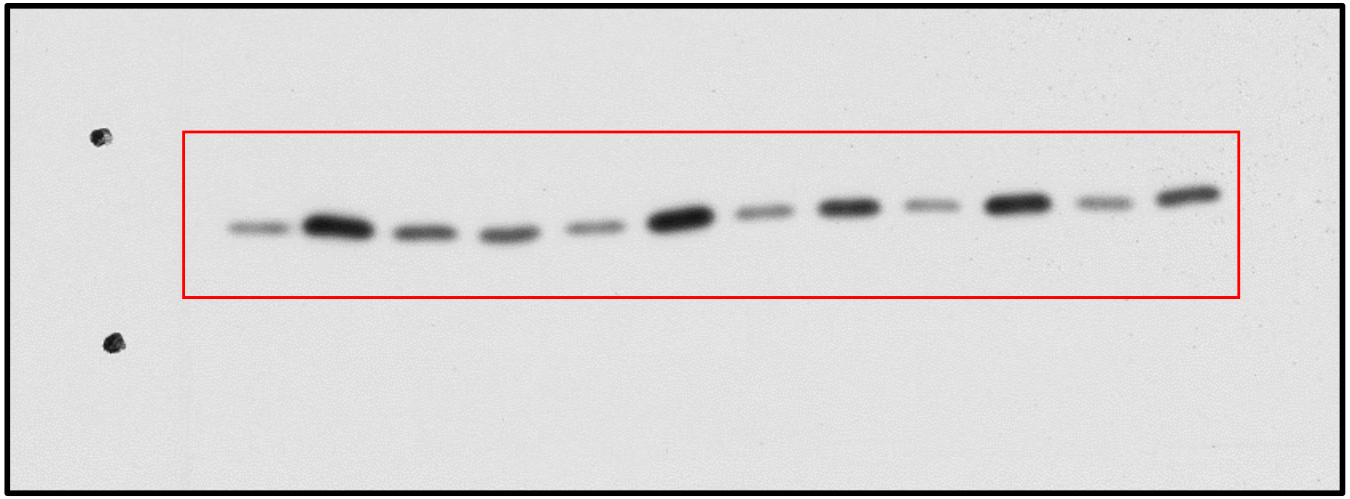

LC3-II

The original full-length images for western blots in Figure 5A

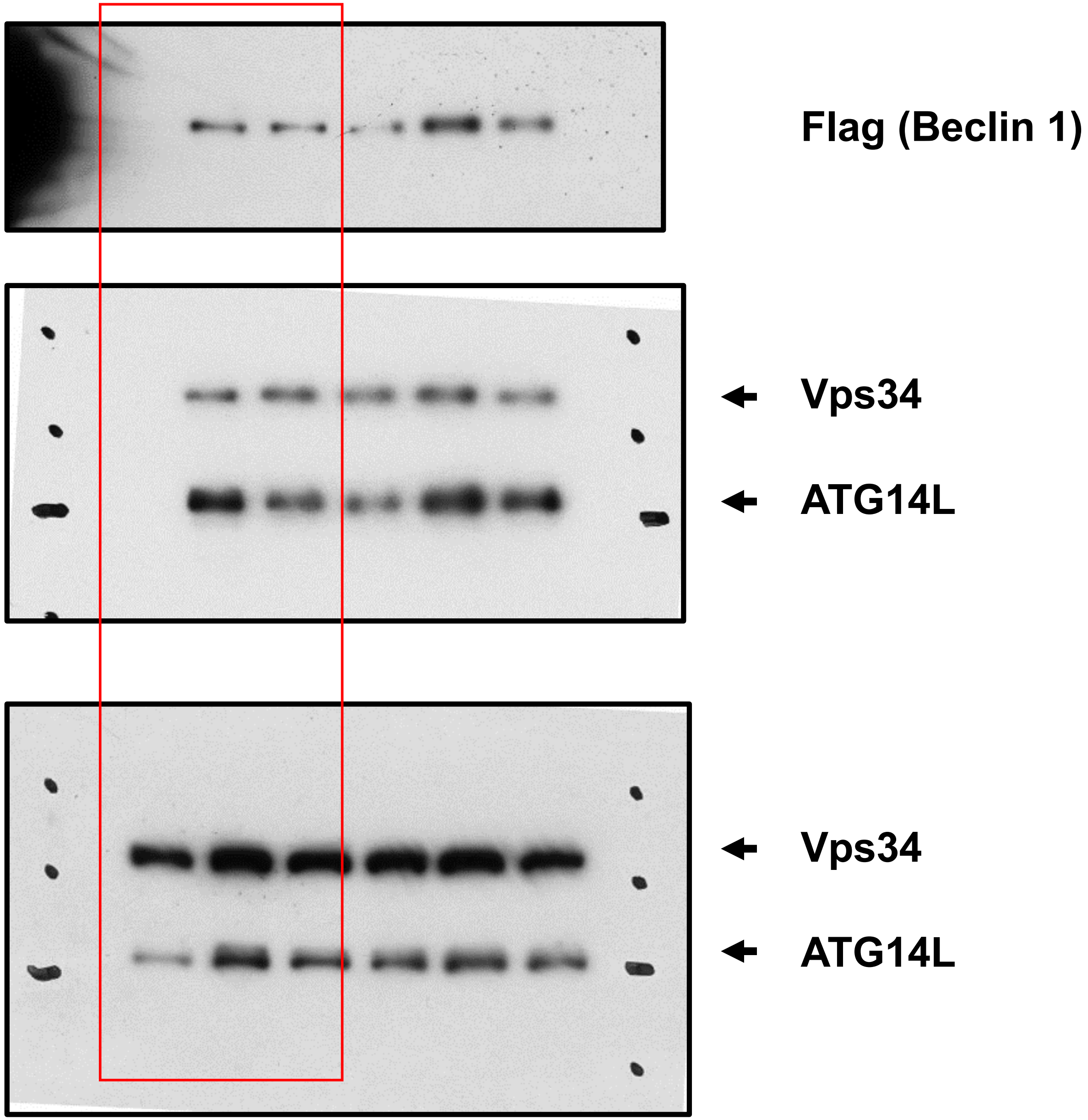

The original full-length images for western blots in Figure 5B

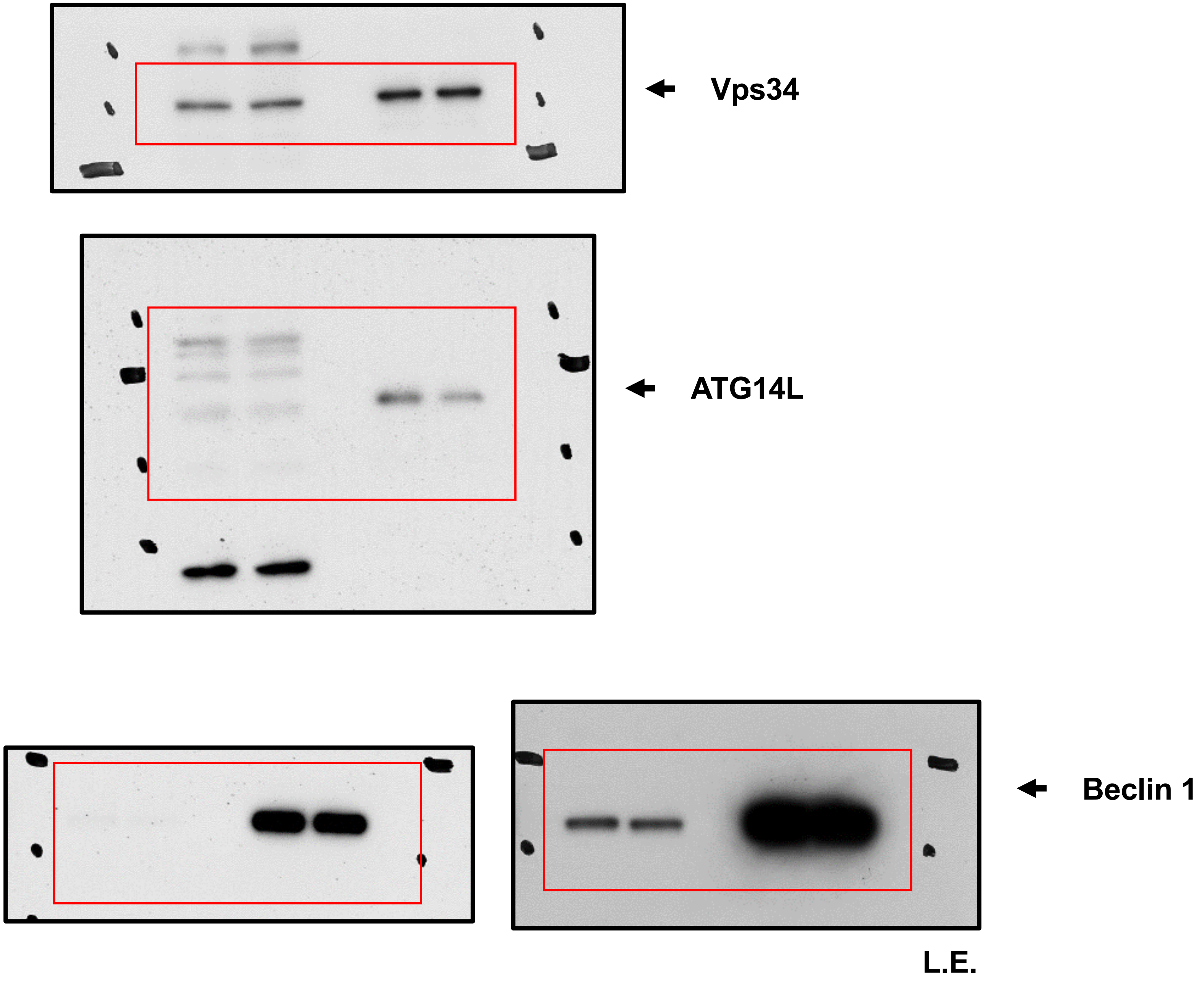

The original full-length images for western blots in Figure 5C

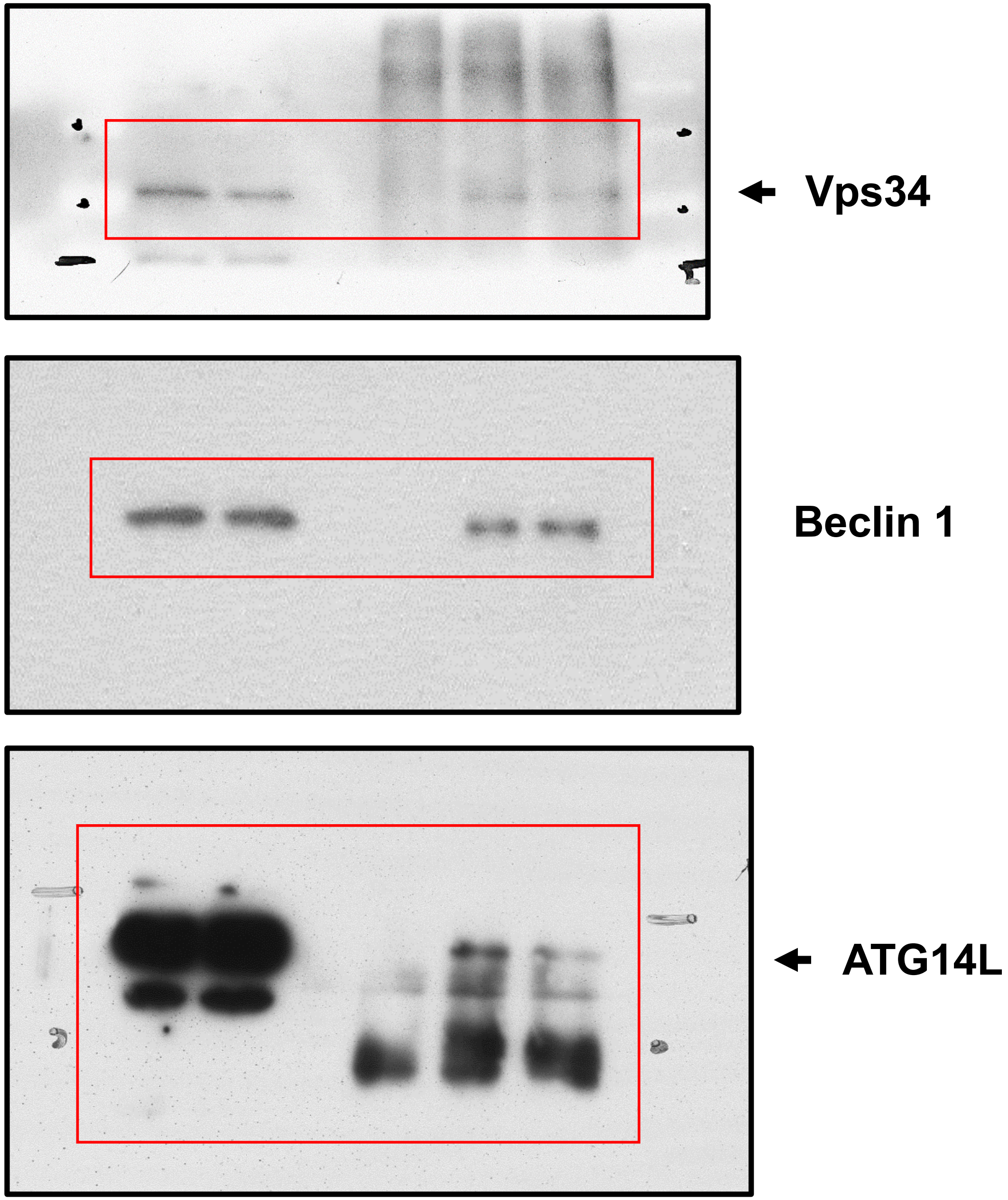

The original full images for western blots in Figure 5D

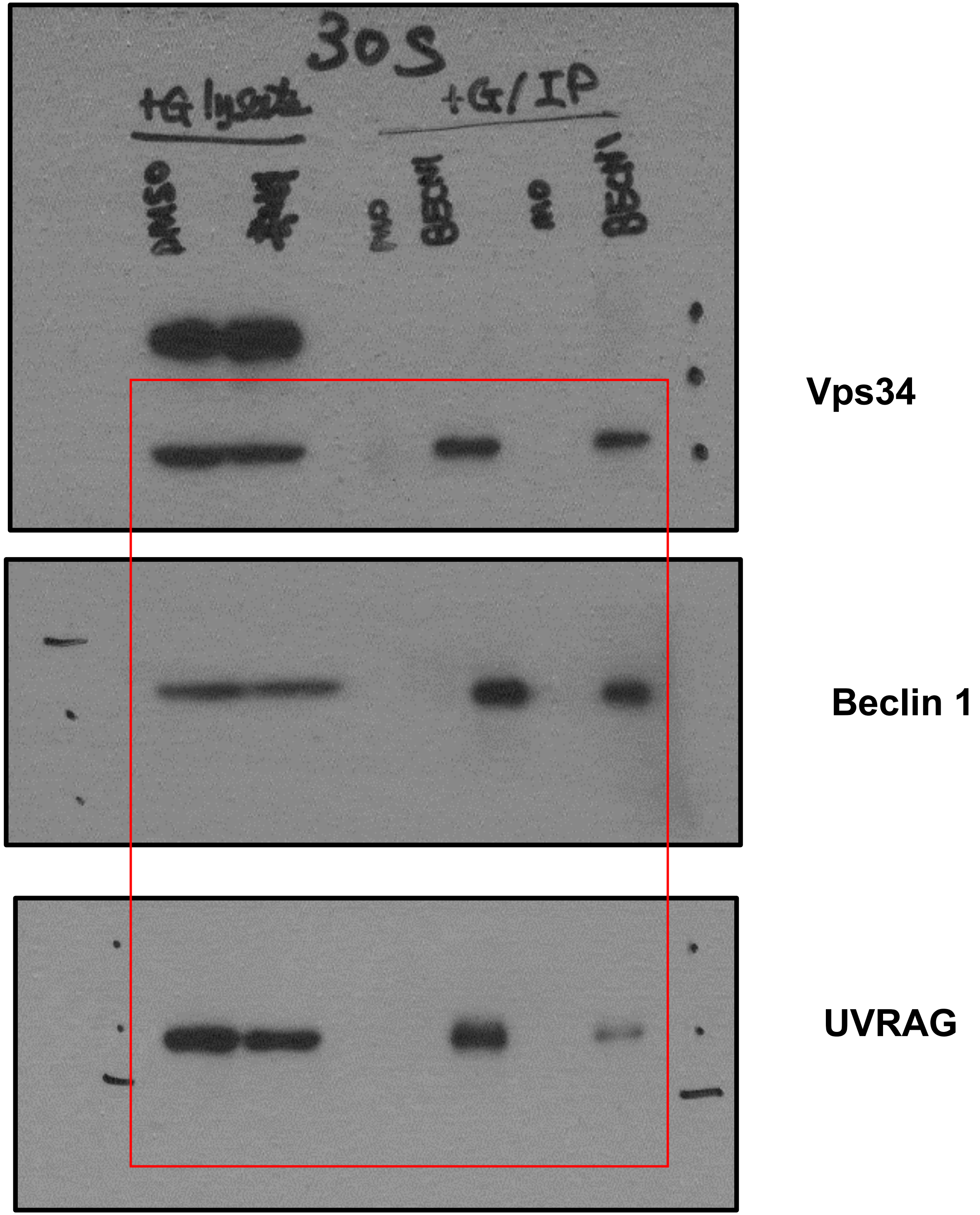

Supplement: Supplementary file 1 — Supplementary Figures. [file 41598_2021_97216_MOESM1_ESM.pdf]
